# *Bupleuri Radix* Polysaccharides Alleviate MASLD by Regulating *Muribaculaceae*-Derived SCFAs in the Gut–Liver Axis

**DOI:** 10.3390/ijms27020637

**Published:** 2026-01-08

**Authors:** Yang Yang, Hong Wang, Yiqing Gu, Ruiyu Wu, Wenqing Qin, Ranyun Chen, Guifang Fan, Xiaoyong Xue, Jianhang Lan, Zixi Huang, Qi Han, Runping Liu

**Affiliations:** 1 School of Chinese Materia Medica, Beijing University of Chinese Medicine, 11 Bei San Huan Dong Road, Beijing 100029, China; 2 School of Life Sciences, Beijing University of Chinese Medicine, 11 Bei San Huan Dong Road, Beijing 100029, China

**Keywords:** *Bupleuri radix* polysaccharides, MASLD, *Muribaculaceae*, *Paramuribaculum intestinale*, SCFAs

## Abstract

*Bupleuri radix* has demonstrated therapeutic potential in treating liver disorders, and polysaccharides are one of its main bioactive components; however, the effects of *Bupleuri radix* polysaccharides (BRP) on metabolic dysfunction-associated steatotic liver disease (MASLD) remain unclear. This study aimed to identify the BRP fractions with anti-MASLD activity and elucidate their underlying mechanisms. We prepared BRP and characterized its physicochemical properties. It markedly alleviated liver injury and restored intestinal barrier function in MASLD. The correlation analysis between transcriptomics and targeted metabolomics showed that BRP restored intestinal acetic acid and propionic acid, with acetic acid activating AMPK and propionic acid promoting cholesterol efflux and metabolism in the liver, thereby reducing lipid accumulation in hepatocytes. Mechanistically, 16S RNA sequencing and diversity analysis indicated that BRP enriched short chain fatty acids (SCFAs)-producing bacteria, such as the genus *Muribaculaceae*, and inhibited pro-inflammatory microbiota. Interestingly, *Paramuribaculum intestinale* (*P. intestinale*), a representative species in the genus *Muribaculaceae*, synergistically enhanced BRP in improving liver and colonic mucosal damage in MASLD. In conclusion, our findings revealed that BRP improved MASLD by regulating *Muribaculaceae*-derived SCFAs in the gut–liver axis and could be used in combination with probiotics as a novel therapeutic strategy for MASLD.

## 1. Introduction

Metabolic dysfunction-associated steatotic liver disease (MASLD) is the most common chronic liver disease worldwide, with an overall prevalence of approximately 25% in the adult population, and even 50% to 70% in obese, type 2 diabetes or metabolic syndrome patients [1,2]. MASLD is not only an important precursor state for liver cirrhosis and hepatocellular carcinoma but is also closely associated with an increased risk of extrahepatic complications such as cardiovascular events and chronic kidney disease, imposing a heavy burden on public health and health-care systems [3,4].

The pathological progression of MASLD is generally considered a multifactorial process, involving excess energy, mitochondrial dysfunction, oxidative stress, lipotoxicity, and chronic inflammation [5,6]. In particular, high-fat diets and metabolic disturbances disrupt intestinal barrier function and promote gut microbiota dysbiosis, allowing bacterial endotoxins to reach the liver and perpetuate a fibro-inflammatory cycle [7]. Moreover, the dysbiosis reduces the generation of beneficial microbial metabolites such as short-chain fatty acids (SCFAs), which typically inhibit inflammatory signaling, maintain the integrity of the intestinal–liver barrier function, and improve the homeostasis of systemic glucose and lipid metabolism [8,9,10,11]. These findings underscore the gut microbiota–liver axis as a pivotal target for therapeutic intervention in MASLD.

Among natural products, plant-derived polysaccharide constituents originating from traditional Chinese medical materials, which are commonly obtained through aqueous extraction, are often collectively referred to as traditional Chinese medicine (TCM) polysaccharides. They have attracted growing attention in MASLD prevention and treatment research due to their high molecular weight, structural diversity, low toxicity, and excellent biocompatibility [12]. Unlike chemically synthesized drugs that target a single pathway, polysaccharides tend to work through multiple targets and pathways in a coordinated way [12,13]. Accumulating evidence indicates that TCM polysaccharides can modulate gut microbial balance and promote SCFAs production, thereby exerting multi-target and multi-mechanism effects on metabolic regulation [14,15,16].

*Bupleuri radix* has been used traditionally and remains in contemporary clinical practice, particularly in TCM and integrative medicine settings, for the management of a range of hepatobiliary disorders. These include MASLD, chronic viral hepatitis, cholestatic liver disease, and certain functional gallbladder disorders [17]. In current clinical practice, *Bupleuri radix* and *Bupleurum*-containing formulas, such as Xiao-Chai-Hu-Tang and Chaihu-Shugan-San, continue to be prescribed for these condition [18]. Their use has been increasingly investigated in modern pharmacological and clinical studies which report effects related to improvement in liver function, regulation of bile acid metabolism, and attenuation of inflammatory and fibrotic responses [19].

Modern pharmacological studies have identified several primary bioactive components in *Bupleurum*, including saponins, flavonoids, volatile oils, and especially polysaccharides [20]. Although our previous studies have demonstrated that saikosaponins are bioactive components with significant regulatory effects on hepatic lipid metabolism [21,22,23], their clinical efficacy may be substantially limited due to poor water solubility, low oral bioavailability, and extensive biotransformation in the gastrointestinal tract [24,25]. In contrast, the water-soluble polysaccharides of *Bupleuri radix* (BRP), which are readily extracted in decoction, display superior solubility and physicochemical stability. These properties suggests that BRP represents an important and previously underexplored component of *Bupleuri radix*, warranting systematic investigation for its potential contribution to the observed metabolic effects in fatty liver diseases models.

This study aimed to establish a stable extraction and preparation method for BRP and systematically evaluate its therapeutic effects against MASLD using a methionine–choline deficient (MCD) diet feeding mouse model. By integrating transcriptomic, microbiome, and targeted SCFAs metabolomic analysis, we highlight the pivotal role of the gut microbiota–SCFAs–host metabolism axis in the therapeutic effects of BRP and identify the underlying specific functional taxa.

## 2. Results

### 2.1. Preparation and Physicochemical Characterization of BRP

BRP was obtained from *Bupleuri radix* via ethanol reflux defatting, hot-water extraction, ethanol precipitation, Sevag deproteinization, D101 decolorization, anion-exchange purification on DEAE-cellulose, and lyophilization, yielding a brown, loose powder (Figure S1A). FT-IR spectra exhibited characteristic polysaccharide bands (Figure S1B): a broad O-H stretching band at ~3230 cm^−1^; absorptions at 1600–1400 cm^−1^ attributable to hydrogen-bonded water/C-H bending; a strong band at ~1019 cm^−1^ assigned to C-O/C-O-C stretching of the sugar ring/glycosidic linkage; and a ring-vibration/C-O-C bending band at ~613 cm^−1^; notably, amide I/II signals (~1650/1540 cm^−1^) were absent. UV-Vis spectra from 200–800 nm showed a smooth baseline without peaks at 260 or 280 nm, consistent with negligible nucleic-acid and protein signatures and indicating high apparent purity (Figure S1C). Scanning electron microscope (SEM) imaging revealed sheet-like, wrinkled aggregates with a rough, porous surface, consistent across magnifications (Figure S1D). High-performance gel permeation chromatography revealed multiple peaks corresponding to the molecular weight distribution of BRP (Figure S1E). Collectively, the spectroscopic evidence supports that the prepared BRP is a polysaccharide with high apparent purity and a loose, porous microstructure, suitable for subsequent biological experiments.

### 2.2. BRP Attenuates MCD Diet-Induced Liver Injury and Metabolic Dysfunction in Mice

**Figure 1:**
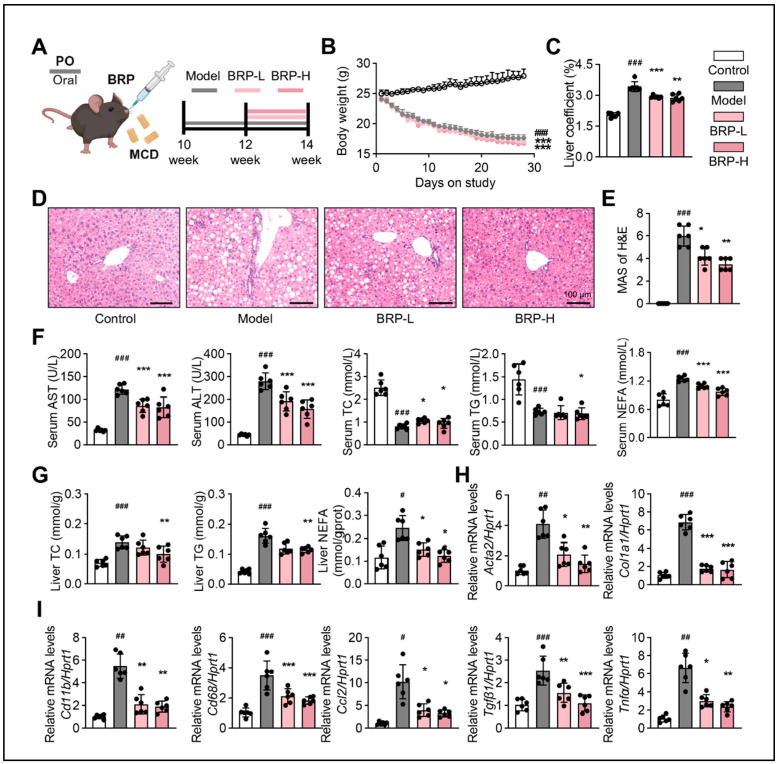
*Bupleuri radix* polysaccharides (BRP) attenuates liver injury and metabolic dysfunction in methionine- and choline-deficient (MCD) diet-induced metabolic dysfunction-associated steatotic liver disease (MASLD) mice. (**A**) Schematic illustration of the experimental design. BRP (200 or 400 mg/kg) was administered to MCD diet-induced mice. (**B**) The body weight of mice in different groups. (**C**) Liver coefficient of mice in each group. (**D**) Hematoxylin and eosin (H&E) staining of liver tissues and (**E**) quantification of the extent of liver damage using the MASLD activity score (MAS) method. Scale bar = 100 μm. (**F**) The levels of aspartate aminotransferase (AST), alanine aminotransferase (ALT), total cholesterol (TC), triglyceride (TG), and non-esterified fatty acids (NEFA) in the serum. (**G**) The levels of TC, TG, and NEFA in the livers. Relative mRNA expression levels of (**H**) fibrosis-related genes *Acta2* and *Col1a1* and (**I**) inflammation-related markers *Cd11b*, *Cd68*, *Ccl2*, *Tgfβ1*, and *Tnfα* in livers. Statistical significance: Data are presented as mean ± standard deviation (SD) (n = 6 mice per group). Statistical analysis was performed using one-way ANOVA followed by appropriate post hoc tests, as described in Section 4. ^#^ *p* < 0.05, ^##^ *p* < 0.01, ^###^ *p* < 0.001 compared between the control (CT) group and the model group. * *p* < 0.05, ** *p* < 0.01, *** *p* < 0.001 compared between the model group and the BRP treatment groups.

To investigate the therapeutic potential of BRP against MASLD progression, ten-week-old C57BL/6 mice were subjected to an MCD diet for four weeks, with BRP administration (200 and 400 mg/kg/day) initiated from the third week (Figure 1A). As expected, MCD feeding led to a progressive reduction in body weight and a significant increase in liver index, whereas BRP treatment markedly attenuated liver enlargement (Figure 1B,C). Histopathological examination by hematoxylin and eosin (H&E) staining demonstrated marked hepatocyte ballooning and vacuolation in the model group, which were significantly attenuated by BRP intervention (Figure 1D,E). Serum biochemical analysis further confirmed hepatic injury in the model group, characterized by elevated aspartate aminotransferase (AST) and alanine aminotransferase (ALT) levels, which were dose-dependently reduced upon BRP administration (Figure 1F). Dysregulated lipid metabolism was evident, with increased hepatic total cholesterol (TC), triglyceride (TG), and non-esterified fatty acid (NEFA) levels alongside decreased serum levels, findings consistent with impaired lipid export. BRP supplementation restored lipid homeostasis, reducing hepatic lipid accumulation and partially normalizing circulating lipid profiles (Figure 1F,G). Furthermore, quantitative real-time PCR (qPCR) revealed that BRP significantly suppressed the upregulation of fibrotic markers (*Acta2*, *Col1a1*, and *Tgfβ1*), macrophage markers (*Cd11b* and *Cd68*), and pro-inflammatory mediators (*Ccl2* and *Tnfα*) (Figure 1H,I). Taken together, these results demonstrated that BRP ameliorated MCD diet-induced hepatic lipid steatosis, fibrosis, and inflammatory responses, suggesting its potential as a therapeutic agent for MASLD.

### 2.3. BRP Alleviates Colonic Mucosal Injury and Inflammation in MCD Diet-Fed Mice

**Figure 2:**
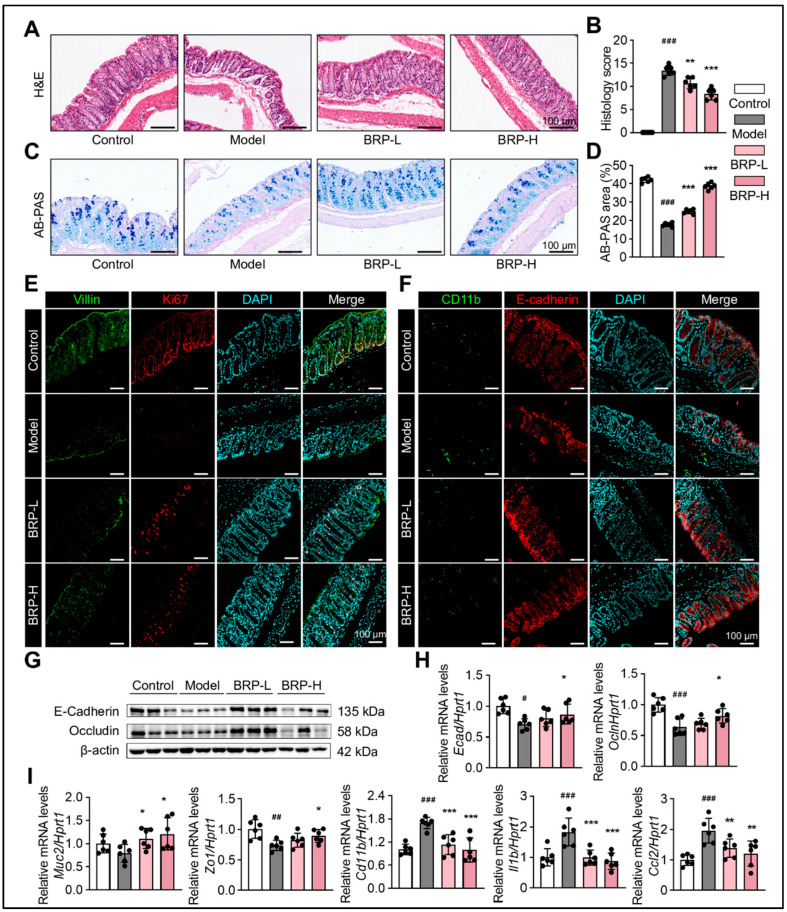
BRP alleviates colonic mucosal injury and inflammation during MASLD. (**A**–**D**) Representative (**A**) H&E staining and (**C**) AB-PAS staining of colon tissues, and (**B**,**D**) the quantification of colonic injury area was performed using Image J software, version 1.53. In H&E staining, nuclei are stained blue-purple and cytoplasm appears pink; in AB-PAS staining, acidic mucins are stained blue and neutral mucins magenta. Scale bar = 100 μm. Representative immunofluorescence co-staining images of (**E**) Villin (green) and Ki67 (red), as well as (**F**) Cd11b (green) and E-cadherin (red), in colon tissue. DAPI was used for nuclear labeling (blue), and yellow signals in merged images indicate colocalization. Scale bar = 100 μm. (**G**) Protein levels of E-cadherin and Occludin in colonic tissues. (**H**,**I**) Relative mRNA levels of *Ecad*, *Ocln*, *Muc2*, *Zo1*, *Cd11b*, *Il1b*, and *Ccl2* in colon tissues. Statistical significance: Data are presented as mean ± SD (n = 6 mice per group). Statistical analysis was performed using one-way ANOVA followed by appropriate post hoc tests, as described in the Section 4. ^#^ *p* < 0.05, ^##^ *p* < 0.01, ^###^ *p* < 0.001 compared between the CT group and the model group. * *p* < 0.05, ** *p* < 0.01, *** *p* < 0.001 compared between the model group and the BRP treatment groups.

As shown in Figure 2, BRP improved intestinal architecture and barrier function in MASLD mice. H&E staining revealed significant epithelial disruption, crypt disorganization, and decreased goblet cell numbers in the model group, while BRP intervention markedly restored tissue morphology (Figure 2A,B). Consistently, Alcian blue-Periodic acid Schiff (AB-PAS) staining demonstrated substantially reduced goblet cell numbers and mucus secretion in the model group, both of which were notably improved by BRP treatment, indicating reinforcement of the mucus barrier (Figure 2C,D). Immunofluorescence staining of Villin and Ki67 showed that compared with the model group, BRP preserved the villus structure and enhanced epithelial proliferation, further confirming the protective effect of BRP (Figure 2E). Furthermore, co-staining of CD11b and E-cadherin showed increased inflammatory cell infiltration and reduced tight junction protein expression in the model group, both of which were significantly ameliorated following BRP treatment (Figure 2F). Moreover, at the protein level, western blotting revealed reduced expression levels of tight junction proteins E-cadherin and Occludin in the model group, which were significantly restored upon BRP supplementation (Figure 2G,H). And at the transcriptional level, the tight junction gene Muc2 and Zo1 were significantly downregulated, while inflammatory mediators such as Cd11b, Ccl2, and Il1b were upregulated in the model group. BRP intervention effectively reversed these alterations, increasing barrier-related gene expression while suppressing inflammatory markers (Figure 2I). Collectively, these findings indicated that BRP ameliorated colonic epithelial disruption in MASLD mice, promoted mucus secretion and epithelial regeneration, and enhanced barrier function through attenuation of inflammation and upregulation of tight junction proteins.

### 2.4. BRP-Driven Transcriptomic Changes Highlight Improved Lipid and Cholesterol Metabolism as Central Pathways in MCD-Fed Mice

**Figure 3:**
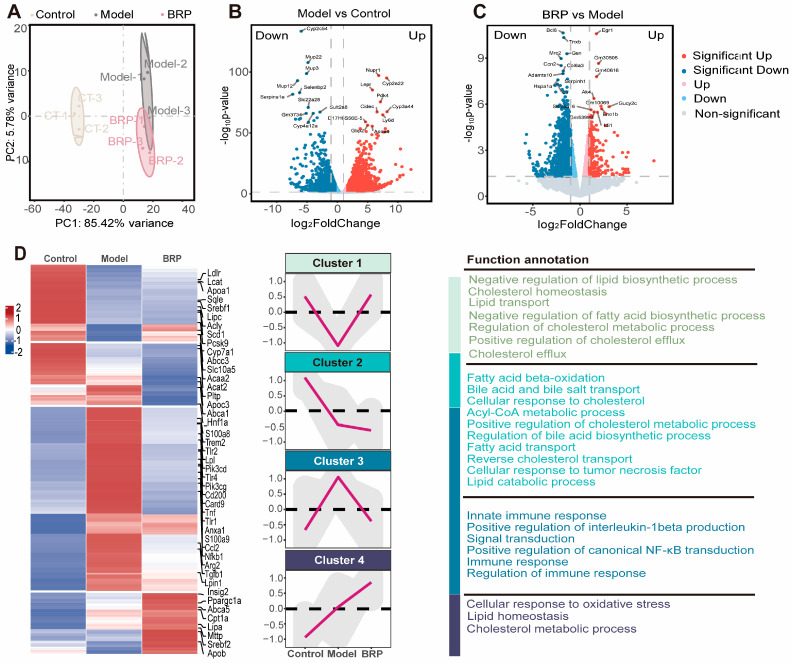

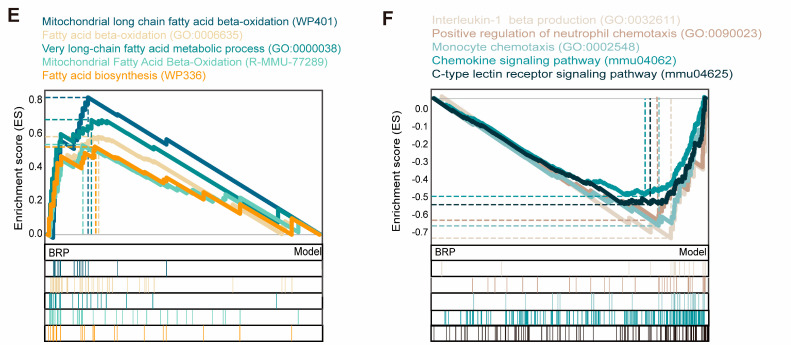
BRP-driven transcriptomic changes highlight improved lipid and cholesterol metabolism in the liver as central pathways during MASLD. (**A**) Principal component analysis (PCA). (**B**,**C**) Volcano plot showed the distribution of differentially expressed genes (**B**) between the CT group and the model group, as well as (**C**) between the model group and the BRP treatment group. (**D**) Z-score heatmap of genes enriched in fatty-acid synthesis/β-oxidation, cholesterol transport/efflux, innate immunity and NF-κB signaling, oxidative stress, and lipid metabolism related GO terms. (**E**,**F**) Gene set enrichment analysis (GSEA) analysis plot showing enrichment of (**E**) fatty-acid β-oxidation, mitochondrial long-chain fatty-acid metabolism, and very-long-chain fatty-acid metabolism, and (**F**) inflammation-related gene sets, such as IL1β production, chemokine signaling pathway, C-type lectin receptor signaling.

To investigate the molecular mechanism by which BRP ameliorates MCD-induced MASLD, we performed transcriptomic profiling of liver tissues from control (CT), MCD, and BRPH-treated mice (Figure 3). Principal component analysis (PCA) showed a clear separation among the three groups, indicating that the MCD diet induced significant alterations in the hepatic transcriptome, while BRP administration substantially reshaped global gene expression patterns (Figure 3A). For example, differentially expressed genes (DEG) analysis revealed that the lipid oxidation regulator Pdk4 was markedly upregulated, while Egr1 expression was suppressed in MCD-fed mice, while BRP treatment reversed these alterations (Figure 3B,C). Further clustering analysis revealed four representative gene expression trajectories. Cluster 1 genes, involved in lipid synthesis suppression, cholesterol transport, and efflux, were downregulated in the model group but restored by BRP, suggesting that BRP reestablishes lipid and cholesterol homeostasis. Cluster 2 genes, enriched in fatty acid β-oxidation and bile acid/cholesterol metabolism, showed a sustained decrease in both the model and BRP groups, implying a compensatory mechanism that restricts lipid overload. Cluster 3 genes, related to innate immune responses, IL1β production, and NF-κB signaling, were markedly induced in the model group but suppressed by BRP. Gene set enrichment analysis (GSEA) supported these findings, showing that pathways associated with fatty acid and cholesterol metabolism were strongly suppressed in the model group but were largely restored after BRP treatment (Figure 3E). At the same time, inflammatory signaling pathways, including chemokine signaling and Il1b production, were strongly enriched in the model group, and this abnormal activation was substantially reduced by BRP intervention (Figure 3F). In summary, rebalancing lipid metabolism together with the alleviation of inflammation might be the central mechanisms underlying the hepatoprotective effects of BRP.

### 2.5. Gut Microbiota-Derived Acetate and Propionate Mediate the Protective Effects of BRP

**Figure 4:**
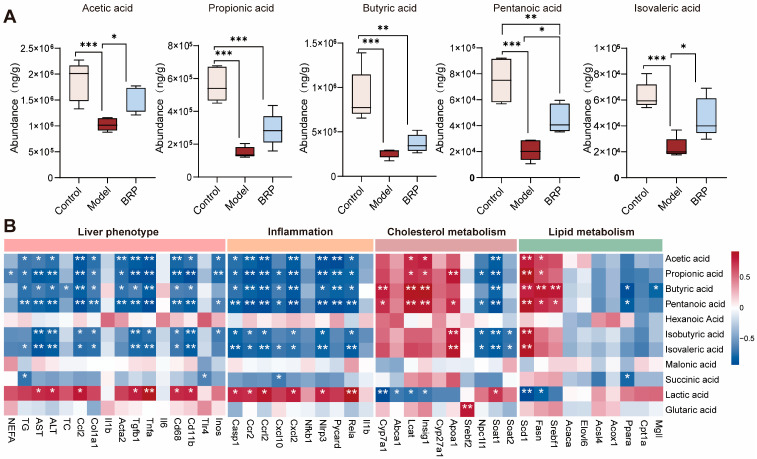
Cecal short chain fatty acids (SCFAs) and their correlations with liver injury, inflammation, and cholesterol/lipid metabolism. (**A**) Abundances of SCFAs (acetic, propionic, butyric, pentanoic, and isovaleric acids) in different groups (*n* = 6). (**B**) Correlation heatmap between cecal SCFAs and liver phenotype (NEFA, TG, ALT, and AST), inflammation (*Ccl2* and *Il1b*), cholesterol metabolism (*Srebf2*, *Hmgcr*, and *Cyp7a1*), or lipid metabolism (*Ppara*, *Cpt1a*, *Srebf1*, and *Fasn*). Color scale denotes Spearman’s ρ (blue, negative; red, positive; range −0.5 to 0.5). Statistical significance: (**A**) Data are presented as mean ± SD, and statistical analysis was performed using one-way ANOVA followed by appropriate post hoc test, as described in Section 4. * *p* < 0.05, ** *p* < 0.01, *** *p* < 0.001 compared between compared between the two indicated groups. (**B**) Correlations were assessed using Spearman’s correlation analysis with false discovery rate (FDR) correction. * *p* < 0.05, ** *p* < 0.01 compared between the model group and the BRP-treatment groups.

Given that BRP is the polysaccharide readily fermented by the gut microbiota and that its main products, SCFAs, play central roles in lipid and cholesterol regulation [8,26], we next performed targeted metabolomic profiling of fecal SCFAs to investigate their potential contribution to the protective effects of BRP. As shown in Figure 4A, MASLD markedly reduced the levels of major SCFAs, whereas BRP intervention restored their abundance. Notably, acetic and propionic acids emerged as the most abundant metabolites, implicating them as the principal effectors through which BRP exerts its protective activity. Correlation analysis further demonstrated that acetic acid and propionic acid levels were negatively associated with the serum levels of AST and ALT, as well as the mRNA levels of inflammatory markers in the liver, such as *Tnfa*, *Ccl2*, *Cd68*, and *Cd11b*. In contrast, these SCFAs showed positive correlations with genes involved in cholesterol transport like *Apoa1* and fatty acid oxidation such as *Acox1*, *Ppara*, suggesting a contribution to enhanced cholesterol efflux and lipid catabolism. Notably, cholesterol synthesis-related genes (*Srebf2*, *Hmgcr*, and *Pcsk9*) also displayed positive associations with SCFAs levels, implying that acetic acid and propionic acid may help rebalance cholesterol synthesis and export to maintain hepatic cholesterol homeostasis (Figure 4B).

**Figure 5:**
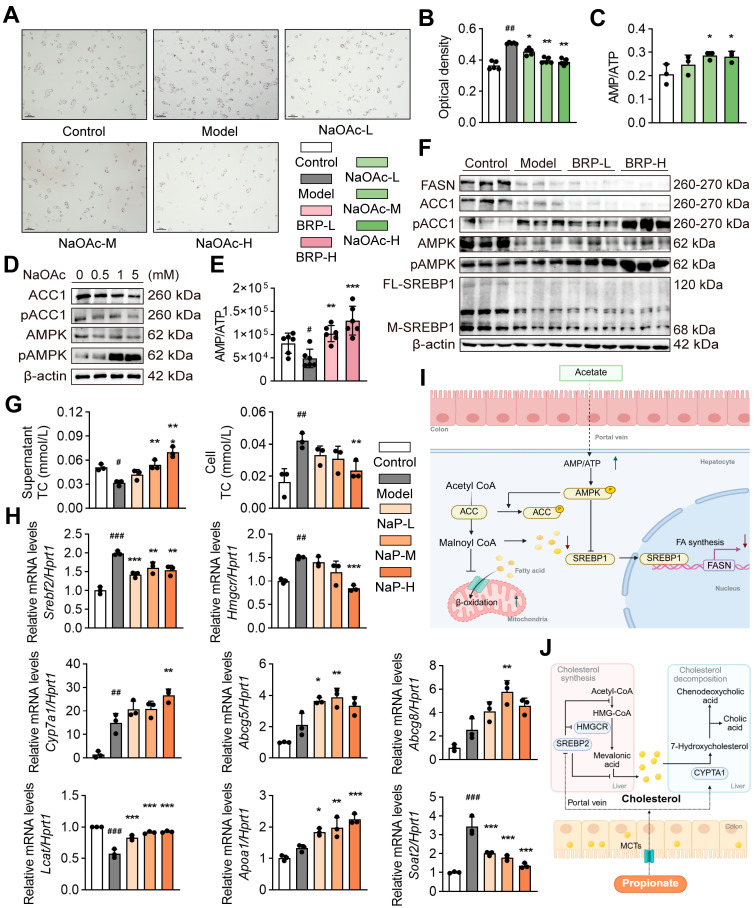
Acetic acid and propionic acid reduce lipid accumulation in liver cells by regulating the cholesterol/lipid metabolism. network. (**A**) Oil Red O staining in AML12 cells. Scale bar = 100 μm. (**B**) Quantification of Oil Red O absorbance. (**C**) The AMP/ATP ratio in AML12 cells. (**D**) The protein levels of AMPK, p-AMPK, ACC1, and p-ACC1 in AML12 cells. (**E**) Hepatic AMP/ATP ratio in mice. (**F**) The protein levels of FASN, ACC1, p-ACC1, AMPK, p-AMPK, and full-length/mature SREBP1 (FL-SREBP1, M-SREBP1) in livers. (**G**) The effect of propionic acid on cholesterol in AML12 cells. Total cholesterol (T-CHO) in the culture supernatant (**left**) and intracellular (**right**). (**H**) Relative mRNA expression levels of *Srebf2*, *Hmgcr*, *Cyp7a1*, *Abcg5*, *Abcg8*, *Lcat*, *Apoa1*, and *Soat2* in AML12 cells. (**I**) Schematic of the acetic acid–AMPK pathway. (**J**) Schematic of the propionic acid–cholesterol pathway. Statistical significance: Data are presented as mean ± SD. Statistical analysis was performed using one-way ANOVA followed by appropriate post hoc tests, as described in the Section 4. *n* = 4 per group for in vitro experiments, and *n* = 6 per group for in vivo experiments. ^#^ *p* < 0.05, ^##^ *p* < 0.01, ^###^ *p* < 0.001 compared between the CT group and the model group. * *p* < 0.05, ** *p* < 0.01, *** *p* < 0.001 compared between the model group and the treatment group.

To verify the role of acetic acid and propionic acid in the protective effects of BRP, we conducted both in vitro and in vivo experiments. We selected AMPK signaling for in vitro validation because AMPK serves as a key metabolic hub linking cellular energy status to fatty-acid oxidation and lipid synthesis. Transcriptomic enrichment and previous literature have both identified AMPK as a core regulator of hepatic energy metabolism and lipid homeostasis. In the AML12 cells exposed to the MCD medium, supplementation with sodium acetate significantly reduced intracellular lipid accumulation as shown by Oil Red O staining (Figure 5A,B). Sodium acetate also activated the AMPK/ACC pathway, evidenced by an increase in the p-AMPK/AMPK ratio and enhanced ACC phosphorylation (Figure 5C). Consistently, acetate treatment elevated the cellular AMP/ATP ratio, and BRP administration in mice produced a similar increase in hepatic AMP/ATP levels, suggesting that BRP promotes fatty acid oxidation and inhibits lipid synthesis through AMPK activation (Figure 5D–F). On the other hand, sodium propionate treatment significantly reduced intracellular cholesterol levels in AML12 cells while increasing cholesterol content in the culture supernatant, indicating enhanced cholesterol efflux (Figure 5G). qPCR analysis further showed that sodium propionate suppressed cholesterol synthesis genes (*Srebf2* and *Hmgcr*) while upregulating genes related to cholesterol export and reverse transport (*Abcg5*, *Abcg8*, *Apoa1*, and *Lcat*), as well as bile acid synthesis mediated by *Cyp7a1* (Figure 5H). Similar results were found in the liver of MASLD mice (Figure S2). Taken together, both SCFAs metabolomics and functional assays demonstrate that BRP enhances acetic acid and propionic acid production in the gut, which in turn activate the AMPK/ACC pathway, inhibit the lipid synthesis, and promote the cholesterol export and conversion. These effects suggested that BRP exerted multi-target metabolic protection through the gut–liver axis.

### 2.6. BRP Significantly Improves the Diversity and Composition of the Intestinal Microbiota in the MCD-Fed Mice

**Figure 6:**
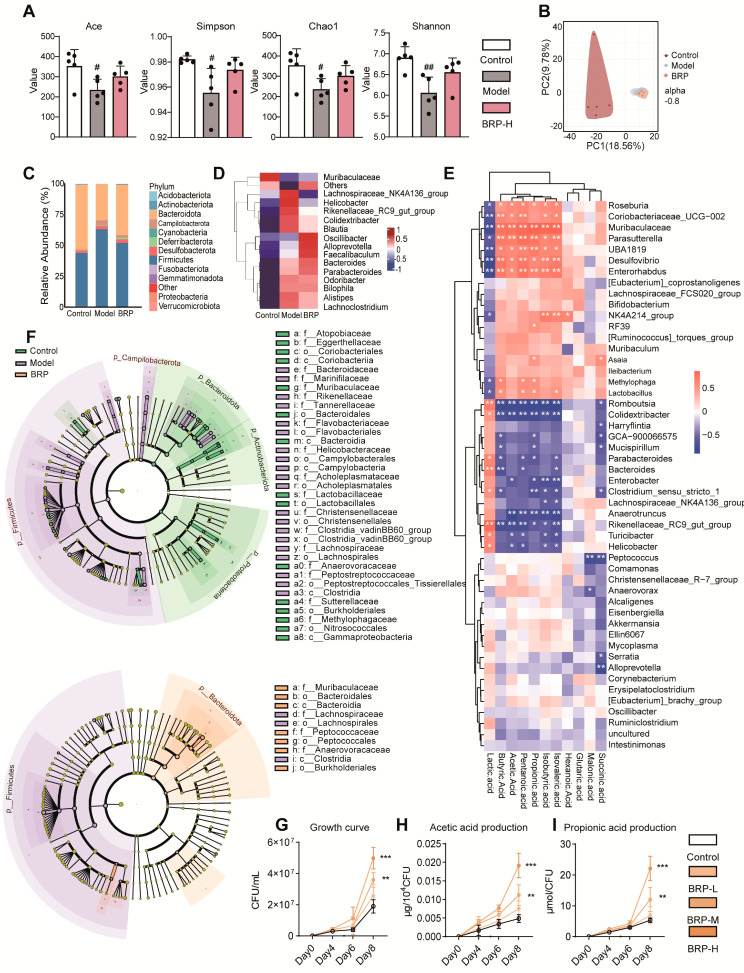
BRP reshapes the gut microbiota and enhances SCFAs production. (**A**,**B**) Microbial diversity was determined by (**A**) the α-diversity, Ace, Simpson, Chao1, and Shannon indices, and (**B**) β-diversity based on PCA. (**C**) Relative abundance of gut microbiota at the phylum level. (**D**) Relative abundance of the top 15 bacteria at the species level. (**E**) The correlation analysis between representative SCFAs and bacteria species. (**F**) The most differentially abundant taxa identified by linear discriminant analysis (LDA) coupled with effect-size measurements (LEfSe) analysis (LDA score ≥ 3.0). (**G**) Growth curves of *P. intestinale*. (**H**) Acetic acid and (**I**) propionic acid production by *P. intestinale.* For panels involving quantitative comparisons, data are presented as mean ± SD (*n* = 6 mice per group), and statistical analysis was performed using one-way ANOVA followed by appropriate post hoc tests, as described in the Section 4. ^#^ *p* < 0.05, ^##^ *p* < 0.01 compared between the CT group and the model group. * *p* < 0.05, ** *p* < 0.01, *** *p* < 0.001 compared between the CT group and the BRP-treatment groups.

Since acetic acid and propionic acid are primarily derived from microbial fermentation of dietary polysaccharides, we hypothesized that the beneficial effects of BRP on hepatic lipid metabolism might depend on its regulation of gut microbiota structure and function. To test this, 16S rRNA sequencing of the cecal contents was performed to evaluate the impact of BRP on intestinal ecology and its relationship with SCFAs production. Alpha-diversity analysis revealed that the model group displayed reduced richness and diversity, with lower ACE, Chao1, Shannon, and Simpson indices, while BRP treatment partially restored these measures (Figure 6A). PCA of β-diversity further showed distinct separation among the three groups, with the BRP group clustering closer to CTs, indicating that BRP mitigated MASLD-induced dysbiosis (Figure 6B). At the phylum level, the MCD diet elevated the *Firmicutes/Bacteroidota* (*F/B*) ratio, a hallmark of dysbiosis associated with metabolic dysregulation [27]. BRP reversed this perturbation, restoring the F/B ratio toward healthy levels (Figure 6C). The heatmap and linear discriminant analysis effect size (LefSe) analysis (Figure 6D–F) consistently showed enrichment of SCFAs-producing genera such as *Alloprevotella* and *Parabacteroides*, after BRP treatment, while pro-inflammatory taxa, including *Bilophila*, *Alistipes*, and *Lachnoclostridium*, were suppressed. These findings indicate a partial reversal of dysbiosis and reinforcement of the SCFAs-producing network [28]. Correlation analysis (Figure 6E) highlighted *Muribaculaceae* as a key responsive taxon to BRP, positively associated with acetic acid and propionic acid. Here, *Muribaculaceae* refers to unclassified genus-level lineages within the *Muribaculaceae* family, consistent with the resolution limit of 16S V3–V4 region sequencing. This taxon is known for polysaccharide fermentation and gut barrier maintenance [29], making it a particularly noteworthy BRP-responsive node within the gut–liver axis.

Since 16S sequencing cannot reliably resolve species-level identities, we focused on well-characterized isolates representing unclassified genus-level members within the *Muribaculaceae* family, including *Paramuribaculum intestinale* (*P. intestinale*) and *Duncaniella muris* (*D. muris*). These two species are the major cultures’ representatives of the *Muribaculaceae* lineage and have been reported to possess well-documented polysaccharide-degrading and SCFAs-producing capacities [30]. Consistently, our 16S analysis showed that the abundance of *Muribaculaceae* increased after BRP intervention, providing the rationale for selecting *P. intestinale* for functional validation as the representative SCFAs-producing strain.

In vitro experiments with graded BRP concentrations (0, 5, 10, 20 mg/mL) showed that BRP promoted the growth of both strains (Figure 6G–I). *P. intestinale* responded more strongly, entering the exponential phase earlier and reaching peak abundance at 10 and 20 mg/mL on days 6 and 8, accompanied by elevated acetate and propionate production (Figure 6G). These outputs remained significant after normalization to CFU, suggesting BRP enhanced not only proliferation but also per-cell SCFAs production (Figure 6H,I). *D. muris* also showed improved activity but to a lesser extent than *P. intestinale* (Figure S3A–C). Collectively, these results demonstrated that BRP acted as a selective growth substrate for *Muribaculaceae*, with *P. intestinale* displaying the strongest proliferative and SCFAs-generating response among representative species, thereby justifying its selection over *D. muris* for subsequent in vivo colonization experiments to functionally validate the role of *Muribaculaceae* in the BRP–gut–liver axis.

### 2.7. Antibiotic-Mediated Microbiota Depletion Abolishes the Hepatoprotective Effects of BRP

**Figure 7:**
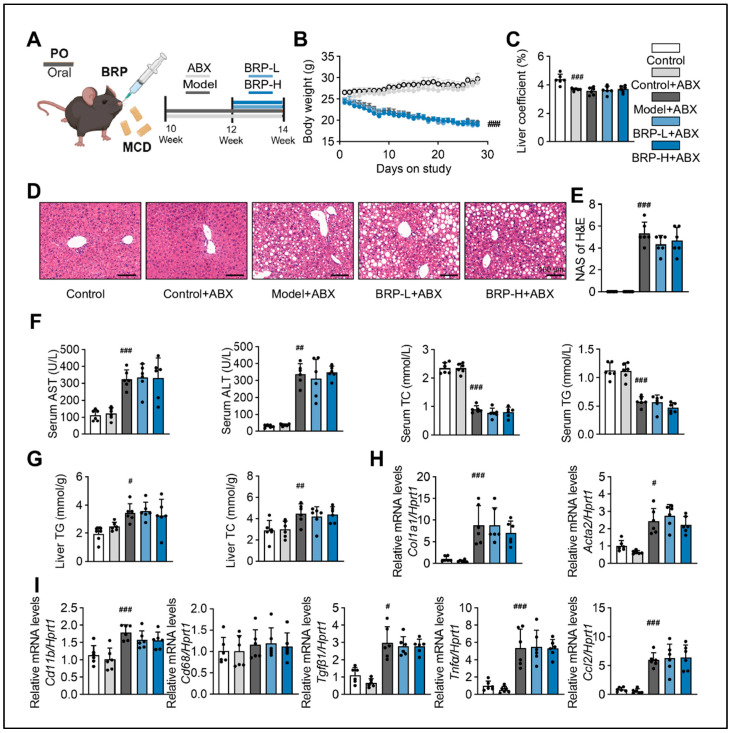
The hepatoprotective effect of BRP is suppressed by removing gut microbiota in MASLD mice. (**A**) Animal experimental flowchart. (**B**) The body weight of mice in different groups. (**C**) Liver coefficient of mice in each group. (**D**) H&E staining of liver tissues, and (**E**) quantification of the extent of liver damage using the MAS method. Scale bar = 100 μm. (**F**) The levels of AST, ALT, TC, and TG in the serum. (**G**) The levels of TG and TC in the livers. (**H**,**I**) Relative mRNA expression levels of *Col1a1*, *Acta2*, *Cd11b*, *Cd68*, *Tgfβ1*, *Tnfα*, and *Ccl2* in livers. Statistical significance: Data are presented as mean ± SD. Statistical analysis was performed using one-way ANOVA followed by appropriate post hoc tests, as described in the Section 4. *n* = 6 mice per group. ^#^ *p* < 0.05, ^##^ *p* < 0.01, ^###^ *p* < 0.001 compared between the CT group and either the CT + ABX group or the model + ABX group.

To determine whether the metabolic protective effects of BRP depend on the intestinal microbiota, we established a mouse model treated with a broad-spectrum antibiotic cocktail to deplete intestinal microbiota, followed by different interventions (Figure 7A). Under antibiotics (ABX) intervention, BRP failed to improve body weight loss or the liver index (Figure 7B,C) in contrast to the effects observed in microbiota-intact mice. Histological examination of liver tissues revealed that MCD + ABX mice still developed marked steatosis and inflammatory infiltration, and BRP administration at either low or high doses did not significantly alleviate these changes. Correspondingly, histological scores showed no difference between groups. (Figure 7D,E). Serum analysis further confirmed that BRP was unable to reduce ALT, AST, or serum TC and TG levels under ABX treatment (Figure 7F), and hepatic TG and TC contents remained unchanged (Figure 7G). At the molecular level, the expression of genes related to fibrosis and inflammation showed no significant differences between the MCD + ABX and BRP + ABX groups (Figure 7H). These findings demonstrate that the protective effects of BRP on liver injury, lipid metabolic disorders, and hepatic inflammation are dependent on regulating gut dysbiosis.

### 2.8. P. intestinale Colonization Partially Recapitulates and Synergizes with the Therapeutic Effects of BRP

**Figure 8:**
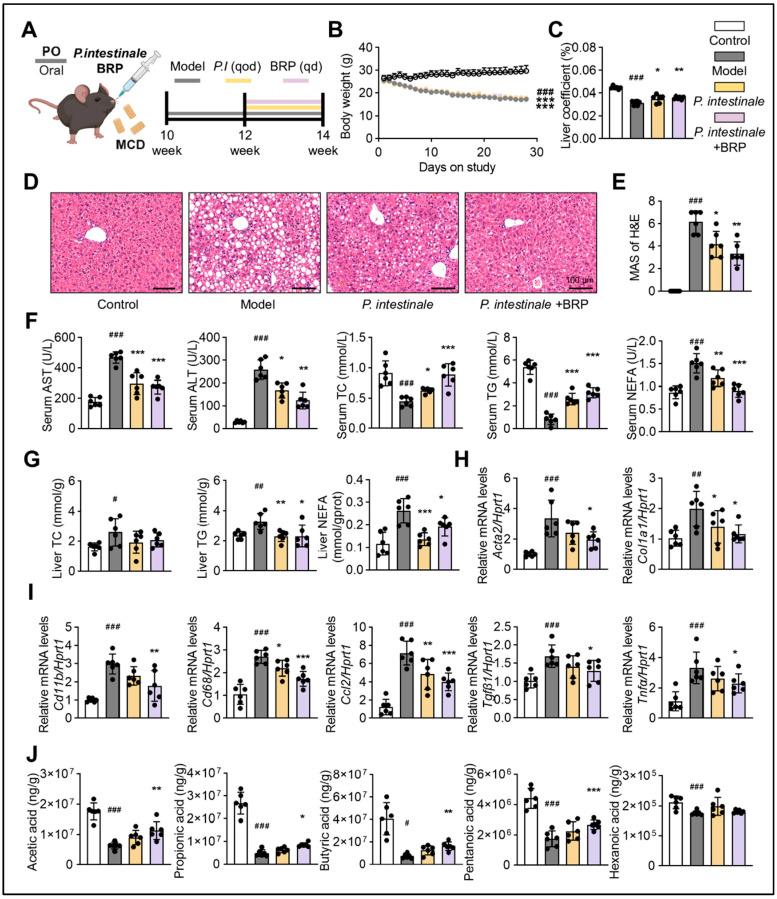
*P. intestinale* synergistically enhances the hepatoprotective effect of BRP on MALFD mice. (**A**) Animal experimental flowchart. (**B**) The body weight of mice in different groups. (**C**) Liver coefficient of mice in each group. (**D**) H&E staining of liver tissues and (**E**) quantification of the extent of liver damage using the MAS method. Scale bar = 100 μm. (**F**) The levels of AST, ALT, TC, TG, and NEFA in the serum. (**G**) The levels of TC, TG, and NEFA in the livers. (**H**,**I**) Relative mRNA expression levels of *Acta2*, *Col1a1*, *Cd11b*, *Cd68*, *Ccl2*, *Tgfβ1*, and *Tnfα* in livers. (**J**) The levels of SCFAs (acetic, propionic, butyric, pentanoic, and isovaleric acids) in cecum. Statistical significance: Data are presented as mean ± SD. Statistical analysis was performed using one-way ANOVA followed by appropriate post hoc tests, as described in the Section 4. *n* = 6 mice per group. ^#^ *p* < 0.05, ^##^ *p* < 0.01, ^###^ *p* < 0.001 compared between the CT group and the model group. * *p* < 0.05, ** *p* < 0.01, *** *p* < 0.001 compared between the model group and the treatment groups.

To confirm the role of *Muribaculaceae* as a key mediator of the protective effects of BRP, we colonized *P. intestinale* in the MCD-fed mice (Figure 8A). As shown in Figure 8B–D, the colonization with *P. intestinale* partially improved MCD diet-induced phenotypes, including body weight loss, steatosis, and inflammatory infiltration in the liver. The serum levels of NEFA decreased, while serum TG and TC returned toward CT levels, and hepatic TG and TC were reduced. These improvements were most evident in the *P. intestinale* combined with the BRP group, which also showed significantly lower serum AST and ALT (Figure 8E,F). *P. intestinale* colonization also suppressed the expression of fibrosis and inflammation-related genes in the liver (Figure 8G). Importantly, BRP combined with *P. intestinale* produced stronger effects, further improving serum and hepatic metabolic indicators, attenuating inflammation and fibrosis, and significantly increasing cecal acetic acid and propionic acid levels (Figure 8H), suggesting a synergistic interaction.

**Figure 9:**
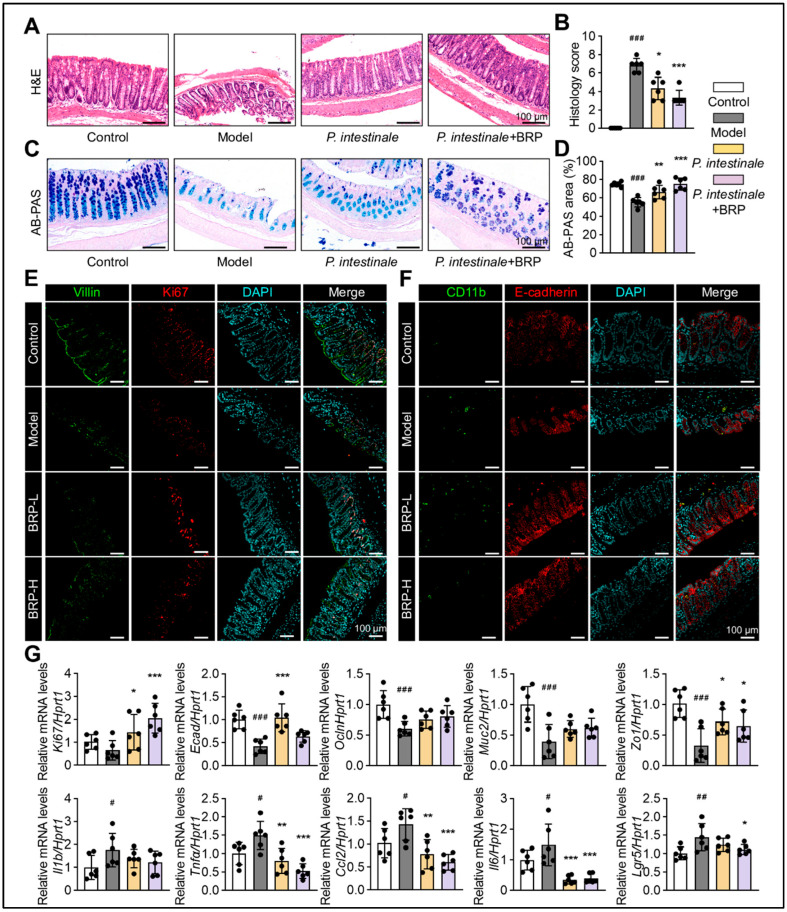
*P. intestinale* synergistically enhances BRP in improving colonic mucosal damage in MALFD mice. (**A**–**D**) Representative (**A**) H&E staining and (**C**) AB-PAS staining of colon tissues, and (**B**,**D**) the quantification of colonic injury area was performed using Image J software. In H&E staining, nuclei are stained blue–purple and cytoplasm appears pink; in AB-PAS staining, acidic mucins are stained blue and neutral mucins magenta. Scale bar = 100 μm. (**E**,**F**) Representative immunofluorescence co-staining images of Villin (green) and Ki67 (red), as well as Cd11b (green) and E-cadherin (red), in colon tissue. DAPI was used for nuclear labeling (blue). Scale bar = 100 μm. (**G**) Relative mRNA levels of barrier genes *Muc2*, *Zo1*, *Ecad*, and *Ocln*, stem/proliferation markers *Lgr5* and *Ki67*, and inflammatory genes *Ccl2*, *Il1b*, *Tnfα*, and *Il6* in colon tissues. Statistical significance: Data are presented as mean ± SD. The *p* values were calculated by one-way ANOVA followed by appropriate post hoc tests, as described in the Section 4. *n* = 6 mice per group. ^#^ *p* < 0.05, ^##^ *p* < 0.01, ^###^ *p* < 0.001 compared between the CT group and the model group. * *p* < 0.05, ** *p* < 0.01, *** *p* < 0.001 compared between the model group and the treatment groups.

Analysis of the same cohort revealed marked improvements in intestinal morphology, as expected (Figure 9). In the model group, the villus structure was disrupted, and goblet cell numbers were reduced, with AB-PAS staining indicating impaired mucus secretion. *P. intestinale* colonization partially restored the mucosal structure and goblet cell abundance, while the BRP combined group showed more pronounced recovery (Figure 9A,B). Dual immunofluorescence staining demonstrated reduced inflammatory infiltration, an enhanced epithelial E-cadherin signal, and increased expression of stem cell and proliferation markers *Lgr5* and *Ki67* in the BRP combined group (Figure 9C). At the transcriptional level, BRP coupling with *P. intestinale* significantly downregulated inflammatory genes, while upregulating mucosal barrier genes (*Muc2*, E-cadherin, *Zo1*, and *Ocln*) and proliferation genes (*Lgr5* and *Ki67*) (Figure 9D). Together, these findings demonstrated that *P. intestinale* colonization could partially mimic the metabolic and barrier-protective effects of BRP, while BRP significantly amplified the therapeutic potential of *P. intestinale*.

## 3. Discussion

Our study confirmed that BRP significantly ameliorates MASLD symptoms and further demonstrated that these effects are mediated by the gut microbiota–SCFAs–host metabolism axis. BRP markedly improved the overall metabolic phenotype in experimental animals by regulating lipid metabolism, promoting cholesterol efflux, and suppressing inflammation. Mechanistically, SCFAs, particularly acetic acid and propionic acid, acted as core mediators that activated AMPK signaling, inhibited cholesterol biosynthesis, and strengthened intestinal barrier integrity. Meanwhile, the gut bacterial strain *P. intestinale*, a member of the genus *Muribaculaceae*, functioned as a key bridge in BRP-driven SCFAs production, working synergistically with BRP. Importantly, BRP used in this study represents a refined total polysaccharide mixture containing multiple subfractions with distinct molecular weights, rather than a single homogeneous macromolecule, which is consistent with the typical chemical characteristic of *Bupleuri radix* polysaccharides.

It has been established that *Bupleurum* contains multiple bioactive components, such as saponins, flavonoids, and polysaccharides, which often exert synergistic pharmacological effects, including anti-inflammatory and immunomodulatory actions [31]. In our previous work, Saikosaponin D (SSd) was identified as a potent PPARα agonist that not only promoted fatty acid oxidation but also induced INSIG1/2 expression, effectively inhibiting the maturation of SREBP-1c. Consequently, SSd improved MASLD via simultaneously suppressing lipid synthesis and enhancing lipid catabolism [21]. In addition, the well-documented immunomodulatory properties of saikosaponins suggest that their hepatoprotective effects may not be limited to direct hepatic metabolic regulation. Emerging concepts such as the liver–spleen axis propose that the spleen can respond to gut microbiota-derived circulating molecules and modulate systemic inflammatory tone, which may indirectly influence liver injury in MASLD [32]. In the context of this study, it can be further speculated that the hepatoprotective effects of *Bupleurum* may result from the synergistic interplay between saponins and polysaccharides. SSd provides rapid hepatoprotective and lipid-lowering effects, while BRP, as a selectively fermentable polysaccharide, continuously generates SCFAs through modulation of the gut microbiota, thus offering more sustained gut–liver axis regulation. This synergy of rapid onset combined with sustained release, integrating PPARα-mediated hepatic metabolic regulation with BRP-induced microecological remodeling, may substantially enhance therapeutic efficacy against MASLD.

As strongly implicated candidate downstream mediators of BRP in treating MASLD, acetic acid and propionic acid may play distinct metabolic roles in the liver. Acetic acid primarily activates AMPK by raising the AMP/ATP ratio, thereby leading to phosphorylation-mediated inactivation of ACC and downregulation of SREBP-1c/FASN expression, which markedly promotes fatty-acid β-oxidation. Propionic acid, once taken up by hepatocytes, suppresses cholesterol-biosynthetic enzymes such as HMG-CoA reductase and may upregulate genes including ABCG5/8 and CYP7A1, thereby facilitating cholesterol efflux and bile acid synthesis. Additionally, in the liver, acetic acid and propionic acid can activate SCFAs receptors such as GPR41/GPR43 and inhibit histone deacetylases, thus attenuating inflammatory signaling and reducing pro-inflammatory mediators including TNFα and IL1β, thereby improving inflammation-related metabolic dysfunction [33,34]. It should be emphasized that these pathways represent a mechanistic interpretation supported by prior frameworks and the phenotypic concordance observed in our study, but they do not yet constitute stringent causal proof that acetate and propionate for the hepatoprotective effects of BRP are sufficient.

Moreover, from the perspective of the liver–gut axis, SCFAs can upregulate tight-junction proteins, promote mucin secretion by goblet cells, and enhance the repair of intestinal epithelial cells mediated by crypt cells. While this study focused on intestinal SCFAs measurements, it is important to note that acetic and propionic acids can be absorbed into the portal vein and delivered to the liver, where they modulate lipid and cholesterol metabolism. These effects could jointly contribute to the therapeutic effects of BRP against MASLD. Nevertheless, whether oral supplementation of acetate and propionate alone or in combination is sufficient to reproduce key BRP-associated endpoints in MCD-fed mice remains to be tested, using dosing regimens that approximate physiologically achievable exposure. Importantly, bolus oral delivery may not fully mimic the spatiotemporal exposure generated by continuous microbial fermentation; formulation, pH, osmolality CTs, and dosing frequency should be carefully considered. However, direct quantification of SCFAs concentrations in portal and hepatic circulation will be required in future work to validate the transport kinetics and tissue distribution of BRP-derived metabolites, which may further clarify using stable-isotope tracers and simultaneous portal and hepatic vein sampling.

From a microecological perspective, *P. intestinale* serves as a functional bridge; although it has been isolated from the gut of mice and its genome has been sequenced, the functional research on this species is still limited, and it has not been systematically classified as a probiotic or pathogenic bacterium [35]. Its potential role in inhibiting pro-inflammatory bacteria through competitive interactions has also not been documented. By contrast, evidence linking the genus *Muribaculaceae* (a family-level taxon often represented as an unclassified genus in 16S datasets) to disease states is more substantial. A high abundance of *Muribaculaceae* is often associated with improved metabolic outcomes and reduced inflammation in obesity, metabolic dysregulation, type 2 diabetes, and inflammatory bowel disease. In MASLD and cirrhosis, however, *Muribaculaceae* abundance is frequently reduced, suggesting an inverse association with liver disease severity [29]. Experimental models further show that *Muribaculaceae* depletion coincides with expansion of pro-inflammatory taxa such as *Desulfovibrionaceae*, *Bilophila*, or *Enterobacteriaceae*, implying potential competition in niche occupancy or metabolite utilization, although these mechanisms require strain- and model-specific validation [36,37,38]. In this study, we speculate that BRP treatment promotes the favorable colonization of *Muribaculaceae* in MCD-fed mice. This enrichment may in turn limit the proliferation of inflammation-associated bacteria, thereby contributing to improved gut health. As members of the *Muribaculaceae* family, both *P. intestinale* and *D. muris* metabolize complex carbohydrates into SCFAs to support intestinal homeostasis [39]. Although *D. muris* exhibits a less-pronounced metabolic improvement compared to *P. intestinale*, it still demonstrates a notable degree of metabolic activity (Figure S3). These findings suggest that the fermentation of BRP into SCFAs may involve multi-strain cooperation that needs to be elucidated. In addition to the gut-microbiota-mediated fermentation pathway, it is also possible that low-molecular-weight fragments or partially degraded products of BRP may be broken down in the gastrointestinal tract and absorbed into the portal circulation, thereby exerting direct metabolic or immunomodulatory effects on the liver. Previous studies have reported that orally administered natural non-starch polysaccharides can be degraded into low-molecular-weight fragments in the digestive tract, and these fragments are capable of entering the bloodstream [40]. Although in the present study antibiotic treatment significantly attenuated the hepatoprotective effects of BRP, strongly supporting a microbiota-dependent mechanism as the primary pathway, the possibility of a fermentation-independent direct effect cannot be completely excluded and warrants further exploration.

Plant acidic polysaccharides rich in glucuronic acid or methyl/acetyl groups, such as citrus pectin, *Ginkgo biloba* leaf polysaccharides, and *Strychnos nux-vomica* root polysaccharides, are commonly used against MASLD through the gut–liver axis. They improve the microbiota, induce SCFAs production, activate FXR/AMPK, inhibit fat production, promote fatty acid oxidation, and alleviate steatosis and inflammation [41,42]. Further evidence indicates that molecular weight or degree of polymerization shapes the physicochemical and biological properties of polysaccharides, such as solubility and the rate and locale of microbial utilization, so different molecular weight ranges may show distinct hepatoprotective effects. In some models, lower molecular weight fractions provide stronger metabolic or anti-inflammatory benefits, and in other contexts, higher molecular weight fractions predominate via viscosity or barrier-related actions, indicating a non-linear, mechanism-dependent relationship [14,43,44]. Consistent with this view, the total polysaccharides of *Bupleuri radix* usually comprise multiple subfractions with differing physicochemical properties; thus, changes in extraction, purification, and characterization methods often yield multi-peak molecular weight profiles and composite mixtures, which may include water-soluble acidic and neutral polysaccharides [45]. Our profiling likewise shows that BRP displays a multi-peak Mw distribution (Figure S1E), indicating that BRP is a refined total polysaccharide mixture composed of several relatively independent components rather than a single uniform macromolecule. This mixed nature likely enables BRP to exert a balanced regulatory effect on both host metabolism and the intestinal barrier.

To obtain structurally defined subfractions suitable for detailed structure–function studies, we further fractionated BRP in follow-up work and isolated a major homogeneous component, which exhibits a single symmetrical Mw peak and well-defined monosaccharide and linkage features. This finding supports the notion that BRP is composed of multiple independent polysaccharide entities, and such heterogeneity may underlie the balanced effects of *Bupleuri* polysaccharides on both metabolic regulation and intestinal barrier protection. However, it remains to be determined which subfractions are acidic versus neutral, their monosaccharide composition and glycosidic linkages, and whether they differ in their ability to be fermented into acetic acid and propionic acid. Future work should combine ion-exchange or size exclusion fractionation with composition and linkage analysis, such as methylation-GC-MS and NMR, followed by in vitro anaerobic fermentation and stable-isotope tracing, to build a causal chain linking polysaccharide structure, microbial metabolism, and host responses.

Notably, BRP shows prebiotic features, promoting Bifidobacterium and Lactobacillus and improving gut community structure [46]. These prebiotic effects are consistent not only with BRP’s ability to improve microbiota composition and enhance SCFAs production in the mouse MASLD model but also suggest its potential applicability in human MASLD. A number of review studies have reported that significant gut microbiota dysbiosis and related metabolic alterations occur in human MASLD, and these changes can influence lipid metabolism, inflammation, and liver function via the gut–liver axis, indicating that the gut microbiota–SCFA–metabolic pathway may play an important role in the disease process [47]. Therefore, as a natural polysaccharide that can sustainably modulate the gut microecology and promote the generation of beneficial metabolites, the prebiotic intervention strategy of BRP may have translational potential as an adjunctive therapy or functional food for MASLD.

However, several challenges remain for clinical translation. Establishing standardized extraction, quantification, and dosing is essential for reproducible clinical application. Moreover, interindividual variability in baseline gut microbiota may lead to heterogeneous fermentation outcomes and metabolic responses to BRP. In addition, the bioavailability and digestive fate of BRP in humans remain to be clarified. Therefore, future studies should evaluate BRP’s efficacy, safety, and optimal dosing in models closer to human pathology or in early clinical settings, in parallel with structure–function analyses, to facilitate its development as a standardized prebiotic or functional intervention for MASLD.

## 4. Materials and Methods

### 4.1. The Preparation of BRP

Powdered *Bupleurum chinense* DC. decoction pieces were first reflux-extracted with anhydrous ethanol (*w*/*v* = 1:20, 100 °C, 2 h) to remove alcohol-soluble constituents. The residue was then twice reflux-extracted with deionized water (*w*/*v* = 1:20, 100 °C, 2 h each). Combined aqueous extracts were concentrated, precipitated with ethanol, redissolved, deproteinized by the Sevag method (chloroform/n-butanol 4:1), and decolorized on pretreated D101 macroporous resin (polysaccharide 2 mg/mL; resin: polysaccharide 40:1, *w*/*w*). The decolorized solution was concentrated and lyophilized to yield BRP. This workflow was adapted from published procedures for acidic water-soluble polysaccharides of *B. chinense* and from macroporous-resin methods for simultaneous decoloration and deproteinization [48].

### 4.2. Animals and Experimental Design

Male C57BL/6J mice (10 weeks old, 20–22 g) were obtained from SPF (Beijing) Biotechnology Co., Ltd., Beijing China. All experiments were conducted at the Animal Research Center of Beijing University of Chinese Medicine in accordance with institutional guidelines for animal care and use (Animal Ethics Approval Number: BUCM-2025032104-1184. Approval Date: 13 December 2020). Mice were housed in a clean-grade environment under controlled conditions (25 °C, 12 h light/dark cycle) with free access to food and water. The diets used in this study included a standard SPF-grade chow diet and a MCD diet. The MCD diet was a commercially available, standardized formulation supplied by Sibeifu (Beijing, China). This diet is otherwise nutritionally comparable to standard chow in terms of carbohydrate, fat, and protein content, except for the absence of methionine and choline. Feeding mice with an MCD diet for 4 weeks induces hepatic steatosis, inflammation, and metabolic dysfunction by impairing very-low-density lipoprotein secretion and lipid export from hepatocytes [49]. The control diet was a nutritionally complete standard chow containing normal levels of methionine and choline, serving as the dietary control for the MCD intervention.

Experiment 1: A total of 24 mice were randomly divided into four groups (*n* = 6 per group): Control (CT), MCD, MCD + BRP low dose (MCD + BRPL, 200 mg/kg/day), and MCD + BRP high dose (MCD + BRPH, 400 mg/kg/day). Mice in the MCD groups were fed an MCD diet for 4 weeks, while the CT group received a normal chow diet. From the beginning of the third week, all mice received daily oral gavage. The BRPL and BRPH groups were administered BRP at the indicated doses, while the CT and MCD groups received an equal volume of deionized water. At the end of the fourth week, mice were sacrificed for sample collection, including blood and tissues.

Experiment 2: A total of 30 mice were randomly assigned to five groups (*n* = 6 per group): CT, CT + antibiotics (CT + ABX), MCD + ABX, MCD + ABX + BRPL, and MCD + ABX + BRPH. Mice in the CT and CT+ ABX groups were fed a normal chow diet, while the other three groups received the MCD diet for 4 weeks. Antibiotics were administered via drinking water to achieve continuous and non-invasive depletion of gut microbiota [50]. Except for the CT group, all other groups were supplied with antibiotic-containing drinking water (ABX: ampicillin 0.5 g/L, vancomycin 0.25 g/L, neomycin sulfate 0.5 g/L, metronidazole 0.5 g/L), which was refreshed every three days throughout the experiment. Drinking water was provided ad libitum, and water consumption was monitored at the cage level during the treatment period; no abnormal reduction in water intake was observed. The effectiveness of antibiotic treatment was assessed at the functional level, as reflected by the attenuation of BRP-mediated metabolic and hepatic protective effects. From the third week, all mice were administered daily oral gavage. Mice in the BRPL and BRPH groups received BRP at 200 mg/kg/day and 400 mg/kg/day, respectively, while the remaining groups received deionized water. Mice were sacrificed at the end of the fourth week for sample collection.

Experiment 3: A total of 24 mice were randomly divided into four groups (*n* = 6 per group): CT, MCD, MCD + *P. intestinale* (MCD + *P. intestinale*), and MCD + BRPL + *P. intestinale*. The CT group received a normal chow diet, while the other three groups were fed the MCD diet for 4 weeks. From the beginning of the third week, the latter two groups were orally gavaged with *P. intestinale* suspension (1 × 10^9^ CFU/mL, 200 μL per mouse). The bacterial solution was administered daily for the first two days of week 3, followed by dosing every other day. In the MCD + BRPL + *P. intestinale* group, BRP (200 mg/kg/day) was gavaged 4 h after bacterial administration. The remaining groups received an equal volume of deionized water. All mice were sacrificed at the end of the fourth week for the collection of blood and tissue samples.

### 4.3. Alcian Blue-Periodic Acid-Schiff (AB-PAS) Staining

Colon tissues were fixed in 4% paraformaldehyde for 24 h, followed by routine dehydration and paraffin embedding. Sections (4 μm) were deparaffinized and rehydrated through a graded ethanol series. AB-PAS staining was performed using a commercial kit (Solarbio, G1285, Beijing, China) strictly according to the manufacturer’s instructions to evaluate goblet cell distribution and mucin secretion. After staining, sections were counterstained, dehydrated, cleared, and mounted for imaging. In the final staining result, acidic mucins appeared blue, and neutral mucins appeared purplish-red, with goblet cells clearly delineated.

Quantitative analysis of AB-PAS staining was performed based on the percentage of positively stained area. For each sample, images were acquired from well-oriented colonic sections under identical imaging conditions. The AB-PAS positive area (blue and purplish-red staining) was measured using ImageJ software (version 1.53, NIH, Bethesda, MD, USA) and normalized to the total mucosal area to calculate the percentage of positive staining. At least 3 non-overlapping fields were analyzed per section, and 1 section was evaluated per animal. All image analyses were conducted in a blinded manner.

### 4.4. Hepatic Lipid Content Measurement

Hepatic TC and TG levels were measured using commercial assay kits (Nanjing Jiancheng Bioengineering Institute, Nanjing, China) following the manufacturer’s protocols (TC kit, Cat. No. A111-1-1; TG kit, Cat. No. A110-1-1). Briefly, liver tissues were homogenized using the extraction solvent specified by the manufacturer. For lipid-rich liver samples derived from the MCD model, anhydrous ethanol was used as the extraction solvent, as recommended by the manufacturer. After centrifugation, the supernatant was collected and subjected to enzymatic colorimetric assays based on the COD-PAP method for TC and the GPO-PAP method for TG. Absorbance was measured using a multifunctional microplate reader, and lipid concentrations were calculated based on standard curves and normalized to liver tissue weight (mmol/g liver tissue).

### 4.5. Serum Biochemical Parameter Analysis

Serum levels of TC, TG, ALT, AST, and NEFAs were measured using assay kits supplied by the Nanjing Jiancheng Bioengineering Institute, China. All procedures strictly followed the manufacturer’s instructions. Key steps included reacting freshly isolated serum samples with corresponding reagents, incubating at the designated temperature and duration, measuring absorbance values for each parameter using a multifunctional microplate reader, and calculating concentrations based on standard curves. Results were expressed in standard units (mmol/L or U/L) for evaluating hepatic function and the degree of lipid metabolism dysregulation.

### 4.6. Immunofluorescence Staining

Paraffin-embedded colon tissue sections (4 μm) were baked at 55 °C for 10 min, deparaffinized in 100% xylene, and rehydrated through a graded ethanol series (100%, 95%, 75%, 50%) for 5 min per step, followed by a ddH_2_O rinse. Sections underwent heat-induced antigen retrieval in pH 8.0, 1 mM antigen retrieval solution at 95–98 °C for 40 min. After cooling to room temperature (RT), sections were transferred to PBS and washed on an orbital shaker for 5 min. Sections were then blocked with 0.2% Triton X-100 + 2.5% BSA + 10% goat serum blocking buffer at RT for 30 min. After blocking, primary antibody cocktails were applied: Villin (Proteintech Group Inc., Rosemont, IL, USA; 66096-1-Ig) + Ki67 (Proteintech Group Inc., Rosemont, IL, USA; 27309-1-AP) or CD11b (Proteintech Group Inc., Rosemont, IL, USA; 66519-1-Ig) + E-cadherin (Cell Signaling Technology, Inc., Danvers, MA, USA; 3195S). Sections were incubated overnight at 4 °C in a humidified chamber. The next day, sections were washed 3× in PBS (5 min per wash), followed by incubation with corresponding fluorescent secondary antibodies (Alexa Fluor 488/594) at RT for 1 h in darkness. After PBS washes, nuclei were counterstained with DAPI before mounting. Images were acquired on a FLUOVIEW FV3000 laser-scanning confocal microscope (Evident, formerly Olympus, Tokyo, Japan).

### 4.7. Oil Red O Staining

After cell treatment, discard the culture medium and gently wash the cells with PBS. Fix the cells with 4% paraformaldehyde at RT for 10 min, then wash twice with PBS. Add 60% isopropanol for pretreatment, incubate at RT for 1 min, and discard. Add filtered Oil Red O working solution and stain for 30 min on a shaker, ensuring full coverage of the cell monolayer. After staining, discard the dye, and gently wash the cells three times with PBS. Observe and photograph under an inverted fluorescence microscope. For quantitative analysis, discard PBS, and add 1 mL of 60% isopropanol containing 4% NP-40 to each well. Shake gently at RT for 5 min to elute the Oil Red O dye. Transfer 100 μL of the eluted solution to a 96-well plate, and measure absorbance at 520 nm using a microplate reader.

### 4.8. ATP Content Determination

ATP levels in cultured cells and liver tissue were measured using a commercial ATP Assay Kit (Beyotime Institute of Biotechnology, Haimen, Jiangsu, China; Cat. No. S0026) strictly according to the manufacturer’s instructions. Briefly, cells or liver tissues were lysed or homogenized using the lysis buffer provided in the kit, followed by centrifugation at 12,000× *g* at 4 °C to obtain supernatants. ATP content was determined using a luciferase-based luminescence assay and measured with a multifunctional microplate reader. ATP concentrations were calculated based on the standard curve.

### 4.9. Measurement of AMP Levels

AMP levels in cultured cells and liver tissues were quantified using a commercial ELISA kit (Shanghai Enzyme-linked Biotechnology Co., Ltd., Shanghai, China; catalog no. ml058338-1) according to the manufacturer’s protocol. Cell lysates or liver tissue homogenates were centrifuged to collect supernatants and subjected to ELISA analysis. Absorbance was measured at 450 nm using a microplate reader, and AMP concentrations were calculated from the standard curve with appropriate dilution factors applied.

### 4.10. Bacterial Culture and BRP Treatment

Blood agar plates were prepared from Columbia blood agar base (BNCC370516, BNCC): the base was dissolved in ultrapure water, autoclaved at 121 °C for 15 min, cooled to 44–47 °C, supplemented aseptically with 5% (*v*/*v*) defibrinated sheep blood (BNCC372938), gently mixed, and poured. For BRP-supplemented plates, a 0.22 µm filtered BRP stock solution was added after blood addition while maintaining 44–47 °C to yield final concentrations of 5, 10, or 20 mg/mL. Suspensions of *P. intestinale* and *Duncaniella muris* (*D. muris*) were evenly spread onto CT and BRP plates and incubated at 37 °C under strict anaerobiosis. Starting from the day of plating, samples were collected on days 4, 6, and 8 for subsequent growth and metabolite analyses.

### 4.11. Measurement of Bacterial Growth Curve

Bacterial biomass was scraped and resuspended in sterile water, mixed thoroughly, and 100 µL was transferred to a 96-well plate for OD_600_ measurement on a microplate reader (diluent as the blank). For convenience, readings were converted to an approximate concentration using the conventional E. coli conversion: OD_600_ = 1 ≈ (0.8–1.0) × 10^9^ CFU/mL.

### 4.12. Acetic Acid Production Assay

Acetic acid production was quantified using a commercial acetic acid assay kit (MLBIO, Cat. No. ml320012), strictly according to the manufacturer’s instructions. Bacterial samples were processed using the extraction buffer provided in the kit, and clarified supernatants were obtained for analysis. Acetate content was determined based on a coupled enzymatic reaction that monitors NADH consumption, with absorbance measured at 340 nm. Acetic acid levels were calculated according to the manufacturer’s formula.

### 4.13. Propionic Acid Production Assay

Propionic acid levels were measured using a commercial ELISA kit (Meiao Biotechnology, Shanghai, China; Cat. No.MO-q91172T) following the manufacturer’s protocol. Clarified bacterial lysates were subjected to ELISA analysis, and absorbance was read at 450 nm using a microplate reader. Propionate concentrations were calculated from the standard curve and adjusted by the corresponding dilution factors.

### 4.14. Statistical Analysis

All data were presented as mean ± SD. Statistical analyses were performed using SPSS software (IBM Corp., Armonk, NY, USA; version 31.0.1). For comparisons among multiple groups, parametric statistical tests were applied. Homogeneity of variances was assessed using Levene’s test, and Welch’s correction was applied when appropriate. When the assumption of variance homogeneity was satisfied, one-way analysis of variance (ANOVA) was used, followed by LSD or Bonferroni post hoc tests for multiple comparisons. When variance homogeneity was not met, Tamhane’s T2 test was applied for post hoc comparisons. The threshold for statistical significance was set at *p* < 0.05. No experimental data points were excluded as outliers.

## 5. Conclusions

This study reveals that BRP confers hepatoprotective effects in experimental MASLD models through a gut microbiota-dependent mechanism. BRP supplementation promotes the colonization of the gut-resident bacterium *P. intestinale*, leading to increased production of SCFAs, especially acetic acid and propionic acid. These microbiota-derived metabolites modulate hepatic lipid and cholesterol metabolism, in part through activation of AMPK signaling and regulation of key metabolic pathways, thereby alleviating hepatic steatosis, inflammation, and metabolic dysfunction. Overall, our findings clarify a mechanistically supported gut–liver pathway underlying the hepatoprotective effects of *Bupleuri radix* and provide an experimental rationale for microbiota-targeted therapeutic strategies in MASLD.

## Data Availability

The data presented in this study are available in the article and its Supplementary Materials. Further inquiries can be directed to the corresponding authors.

## Supplementary Materials

The following supporting information can be downloaded at: https://www.mdpi.com/article/10.3390/ijms27020637/s1.

## Abbreviations

AB-PAS: Alcian blue-Periodic acid Schiff; ABX: antibiotics; ALT: alanine aminotransferase; AST: aspartate aminotransferase; BRP: *Bupleuri radix* polysaccharide; BRPH: BRP high dose; BRPL: BRP low dose; CT: control; *D. muris*: *Duncaniella muris*; DEGs: differentially expressed genes; F/B: *Firmicutes/Bacteroidota*; GSEA: gene set enrichment analysis; H&E: hematoxylin and eosin; LEfSe: linear discriminant analysis effect size; LDA: linear discriminant analysis; MAS: MASLD activity score; MASLD: metabolic dysfunction-associated steatotic liver disease; MCD: methionine- and choline-deficient; NEFAs: non-esterified fatty acids; *P. intestinale*: *Paramuribaculum intestinale*; PCA: principal component analysis; qPCR: quantitative real-time PCR; RT: room temperature; SCFAs: short chain fatty acids; SD: standard deviation; SSd: Saikosaponin D; TC: total cholesterol; TCM: traditional Chinese medicine; TG: triglyceride.
