# Supplementary material for: Bupleuri Radix Polysaccharides Alleviate MASLD by Regulating Muribaculaceae-Derived SCFAs in the Gut–Liver Axis
Source: Int J Mol Sci. 2026 Jan 8;27(2):637. doi: 10.3390/ijms27020637 (PMC12841138; doi:10.3390/ijms27020637)
Supplement: Supplementary file 1 [file ijms-27-00637-s001.zip › ijms-4033881-supplementary.pdf]

1. Supplementary materials and methods

1.1 H&E staining and histopathological scoring

Liver and colon tissues were fixed in 4% paraformaldehyde for 24 hours, dehydrated through a graded ethanol series, cleared in xylene, and embedded in paraffin. Sections were cut at 5 μm thickness. After deparaffinization in xylene and rehydration through a descending ethanol series, sections were routinely stained with Hematoxylin and Eosin (H&E), dehydrated, cleared, and mounted. Stained sections were digitally scanned using a whole-slide scanner (Aperio VERSA, Leica Biosystems). Liver pathology was blindly evaluated using the MASLD Activity Score (MAS) system, assessing three parameters: steatosis, ballooning degeneration, and lobular inflammation. Scoring criteria were as follows: Steatosis score: 0 (<5%), 1 (5%-33%), 2 (34%-66%), 3 (>66%); Lobular inflammation score: 0 (none), 1 (<2 foci per field), 2 (2-4 foci), 3 (>4 foci); Ballooning degeneration score: 0 (none), 1 (few ballooned cells), 2 (many ballooned cells). The total MAS ranged from 0 to 8 points, with higher scores indicating more severe liver injury.

Colon tissues were evaluated using an inflammatory activity scoring system assessing: mucosal architecture integrity, glandular arrangement, goblet cell depletion, and inflammatory cell infiltration. The scoring scale was: 0 points: Normal architecture, no inflammatory cell infiltration; 1 point: Mild epithelial detachment, slight goblet cell reduction, mild infiltration; 2 points: Moderate epithelial damage, disordered gland arrangement, marked goblet cell loss, moderate infiltration; 3 points: Severe architectural disruption, extensive goblet cell depletion, significant inflammatory infiltration extending to the submucosa.

For each animal, at least 3 non-overlapping fields from 1 independent sections were evaluated. Histopathological scoring was performed independently by two investigators who were blinded to the experimental groups, and the final score was determined by averaging the scores from both observers.

1.2 RNA extraction and quantitative real-time PCR (qPCR)

Total RNA was extracted from liver tissues, colonic tissues, and cultured cells using the Trizol method. Briefly, appropriate amounts of tissues or cells were homogenized or lysed in Trizol reagent, incubated at room temperature for 10 minutes, followed by the addition of chloroform. After vigorous mixing and phase separation at room temperature, samples were centrifuged at 12,000 × g for 15 minutes at 4°C. The aqueous phase was collected and mixed with an equal volume of isopropanol to precipitate RNA. After washing the RNA pellet with ethanol, it was air-dried at room temperature and dissolved in preheated RNase-free water. RNA concentration and purity were measured using dual-beam nucleic acid/protein spectrophotometer (Nano-800+, Shanghai Jiapeng Technology Co., Ltd.), ensuring an A260/A280 ratio between 1.8 and 2.0. First-strand cDNA was synthesized using the miRNA 1st Strand cDNA Synthesis Kit (MR101-02; Vazyme, Nanjing, China) according to the manufacturer's instructions. The reaction was conducted at 37°C for 15 minutes and terminated at 98°C for 5 minutes. qPCR was performed using miRNA Universal SYBR qPCR Master Mix (MQ102-02; Vazyme) on a CFX96 Touch Real-Time PCR Detection System configured with a C1000 Touch thermal cycler (Bio-Rad). The thermal cycling conditions were: 95°C for 30 seconds for initial denaturation, followed by 40 cycles of 95°C for 5 seconds and 60°C for 30 seconds. Gene expression levels were normalized to Hprt1 and calculated using the $2^{-\Delta\Delta Ct}$ method.

1.3 Protein extraction and western blot analysis

Liver/colon tissues or cells were homogenized in RIPA lysis buffer for protein extraction. Lysates were centrifuged at 12,000 g (4°C) for 15 min, and supernatants were collected for protein concentration measurement using the bicinchoninic acid (BCA) assay (Beijing Bairuiji Biotechnology Co., Ltd.; Cat. BN27109). Equal amounts of protein samples were mixed with loading buffer, denatured at 100°C for 15 min in a heating block, and separated by SDS-PAGE. Proteins were then transferred onto PVDF membranes. Membranes were blocked with 5% skim milk in TBST at room temperature for 1 hour, followed by incubation with primary antibodies (see below) at

4°C overnight. After TBST washes the next day, membranes were incubated with species-matched secondary antibodies at room temperature for 1 hour. Following additional washes, protein bands were visualized using an enhanced chemiluminescent substrate (E1050, LABLEAD, Beijing, China). Image acquisition and grayscale analysis were performed using ImageJ software, with β-actin serving as the internal control.

Details of primary antibodies are listed below:

E-Cadherin (24E10) Rabbit mAb (CST#3195)

Occludin Rabbit pAb (Proteintech, 27260-1-AP)

ACC1 Monoclonal antibody (Proteintech, 67373-1-Ig)

Phospho-ACC1 (Ser79) Polyclonal antibody (Proteintech, 29119-1-AP)

CPT1A Polyclonal antibody (Proteintech, 15184-1-AP)

SREBF1 Polyclonal antibody (Proteintech, 14088-1-AP)

Rabbit polyclonal to AMPK alpha 1 + AMPK alpha 2 (Abcam, ab131512)

Rabbit polyclonal to AMPK alpha 1 (phospho T183) + AMPK alpha 2 (phospho T172) (Abcam, ab23875)

Fatty Acid Synthase (G-11) (Santn cruz, sc-398559)

1.4 Reference-based transcriptomics data analysis workflow

Following RNA extraction, paired-end sequencing was performed on the Illumina NovaSeq 6000 platform. Each sample generated approximately 44-49 million raw reads, with a total data volume of approximately 6.5-7.2 Gb. Raw data underwent quality control and filtering using the fastp software. The filtered reads were then aligned to the reference genome using HISAT2, and gene expression levels were quantified as both Fragments Per Kilobase of transcript per Million mapped reads and raw counts. Differential expression analysis was conducted using DESeq2. Significantly differentially expressed genes (DEGs) were identified based on the criteria: $p < 0.05$ and absolute $\log_2$ (fold change) > 1. Principal Component Analysis (PCA) and heatmap visualization were performed using R (v4.4.1). Functional

enrichment analysis was carried out based on the GO and KEGG databases. Results were visualized using the R packages clusterProfiler and ggplot2. Furthermore, Gene Set Enrichment Analysis (GSEA v4.3.3) was employed to evaluate the enrichment trends of predefined gene sets across different treatment groups.

1.5 16S RNA sequencing and diversity analysis

DNA extraction and PCR amplification: Total DNA from intestinal samples was extracted using the MagPure Soil DNA LQ Kit (Magan). DNA concentration and purity were detected using NanoDrop 2000 (Thermo Fisher Scientific, USA) and agarose gel electrophoresis. DNA samples were stored at -20°C. Using extracted genomic DNA as template, the bacterial 16S rRNA gene was amplified with barcode primers and Takara Ex Taq high-fidelity enzyme. The targeted region was V3-V4, amplified using universal primers: 343F (5'-TACGGRAGGCAGCAG-3') and 798R (5'-AGGGTATCTAATCCT-3'). Library construction and sequencing: PCR products were detected by agarose gel electrophoresis, purified with AMPure XP beads as templates for secondary PCR amplification. After re-amplification and purification, quantification was performed using Qubit. Pooled libraries underwent paired-end 250-bp sequencing on the Illumina NovaSeq 6000 platform. Library construction and sequencing were completed by Shanghai OE Biotech Co., Ltd. 16S rRNA Diversity Sequencing Analysis Methods: Raw sequencing data in FASTQ format were processed by removing adapter sequences using Cutadapt, followed by denoising, splicing, and chimera removal with DADA2 to obtain a high-quality ASV abundance matrix. Using the QIIME2 (2020.11) platform, representative sequences were selected and annotated against the Silva database (version 138) via the qiime2-feature-classifier plugin for taxonomic classification. Alpha diversity indices (e.g., Chao1, Shannon) were calculated using QIIME2 to evaluate species diversity within samples. For beta diversity analysis, microbial community differences were constructed based on Bray-Curtis distance matrices and visualized through PCoA. Inter-group comparisons were performed using R (v4.4.1), employing non-parametric statistical tests (ANOVA), with Linear

Discriminant Analysis Effect Size (LEfSe) analysis identifying key bacterial genera showing significant differences between groups.

2. Supplementary figures

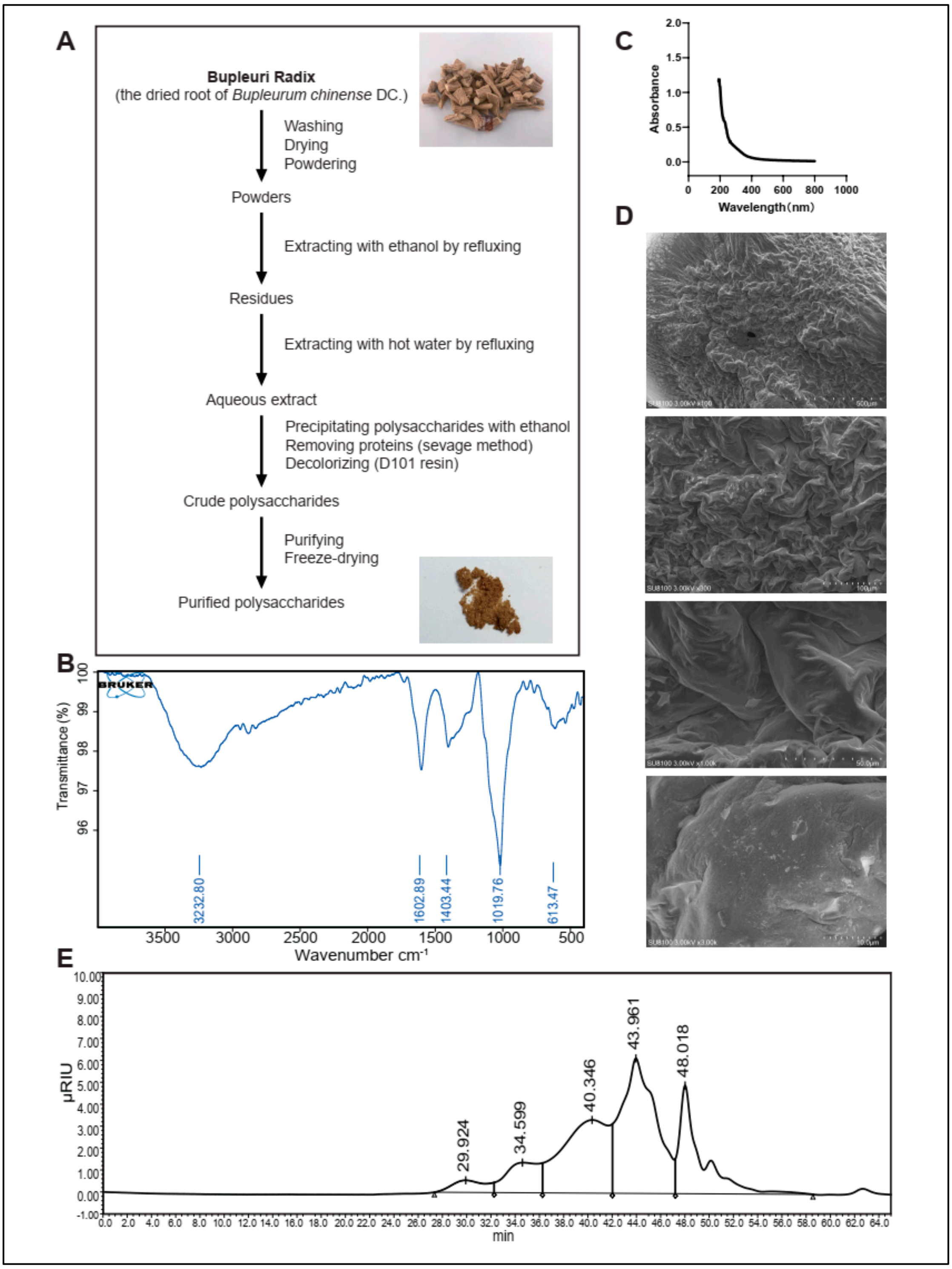

Figure S1. Preparation workflow and physicochemical characterization of BRP. **(A)** Workflow for preparing BRP from *Bupleuri radix*. **(B)** FT-IR spectrum of BRP. **(C)** UV-Vis absorption spectrum of BRP. **(D)** SEM micrographs of BRP. The scale bars from left to right is 10 μm, 50 μm, 100 μm, and 500 μm, respectively. **(E)** Molecular weight distribution of BRP determined by high-performance gel permeation chromatography

(HPGPC).

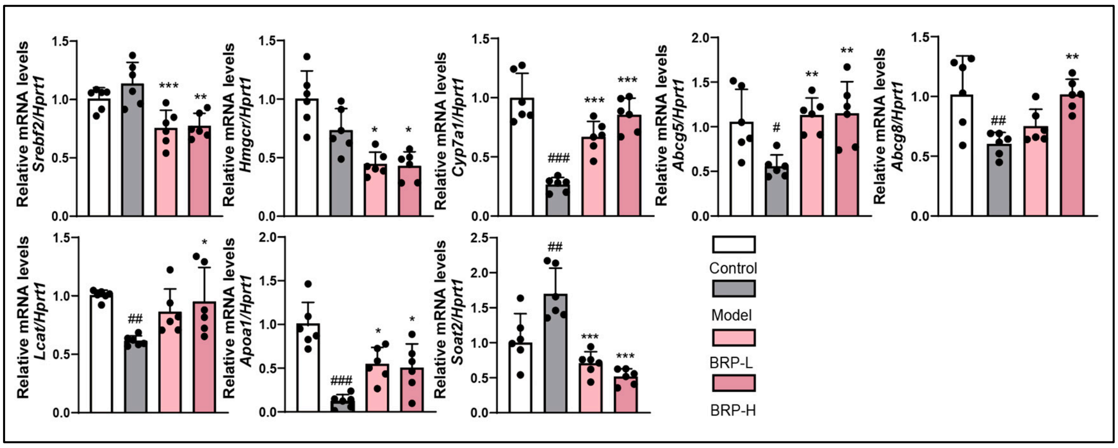

Figure S2. BRP restores the reduced expression of cholesterol metabolism genes in mice with MASLD. Relative mRNA levels of *Srebf2*, *Hmgcr*, *Cyp7a1*, *Abcg5*, *Abcg8*, *Lcat*, *Apoa1*, and *Soat2* in livers. Data are presented as mean ± SD. Statistical analysis was performed using one-way ANOVA followed by appropriate post-hoc tests, as described in the Materials and methods section. $n = 6$ mice per group. $^{\#}p < 0.05$, $^{\#\#}p < 0.01$, $^{\#\#\#}p < 0.001$ compared between the control group and the model group. $^{*}p < 0.05$, $^{**}p < 0.01$, $^{***}p < 0.001$ compared between the model group and the BRP treatment groups.

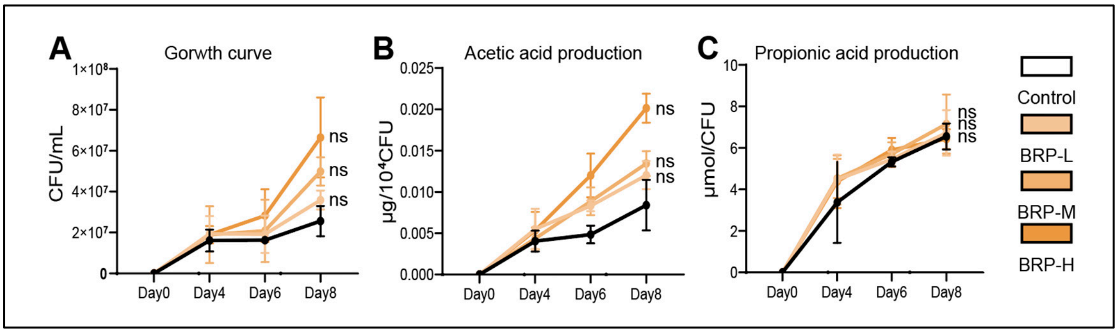

Figure S3. BRP induces cell proliferation and production of SCFAs in *D. muris.* **(A)** Growth curves of *D. muris*. **(B)** Acetic acid and **(C)** propionic acid production by *D. muris.* Data are presented as mean ± SD. Statistical analysis was performed using one-way ANOVA followed by appropriate post-hoc tests, as described in the Materials and methods section. *n* = 6 mice per group. ns. indicates no statistically significant difference compared to the control group.
